# Supplementary material for: Optimizing the Seeding Density of Human Mononuclear Cells to Improve the Purity of Highly Proliferative Mesenchymal Stem Cells
Source: Bioengineering (Basel). 2023 Jan 11;10(1):102. doi: 10.3390/bioengineering10010102 (PMC9855129; doi:10.3390/bioengineering10010102)
Supplement: Supplementary file 1 [file bioengineering-10-00102-s001.zip › bioengineering-2118184-supplementary.pptx]

## Slide 1
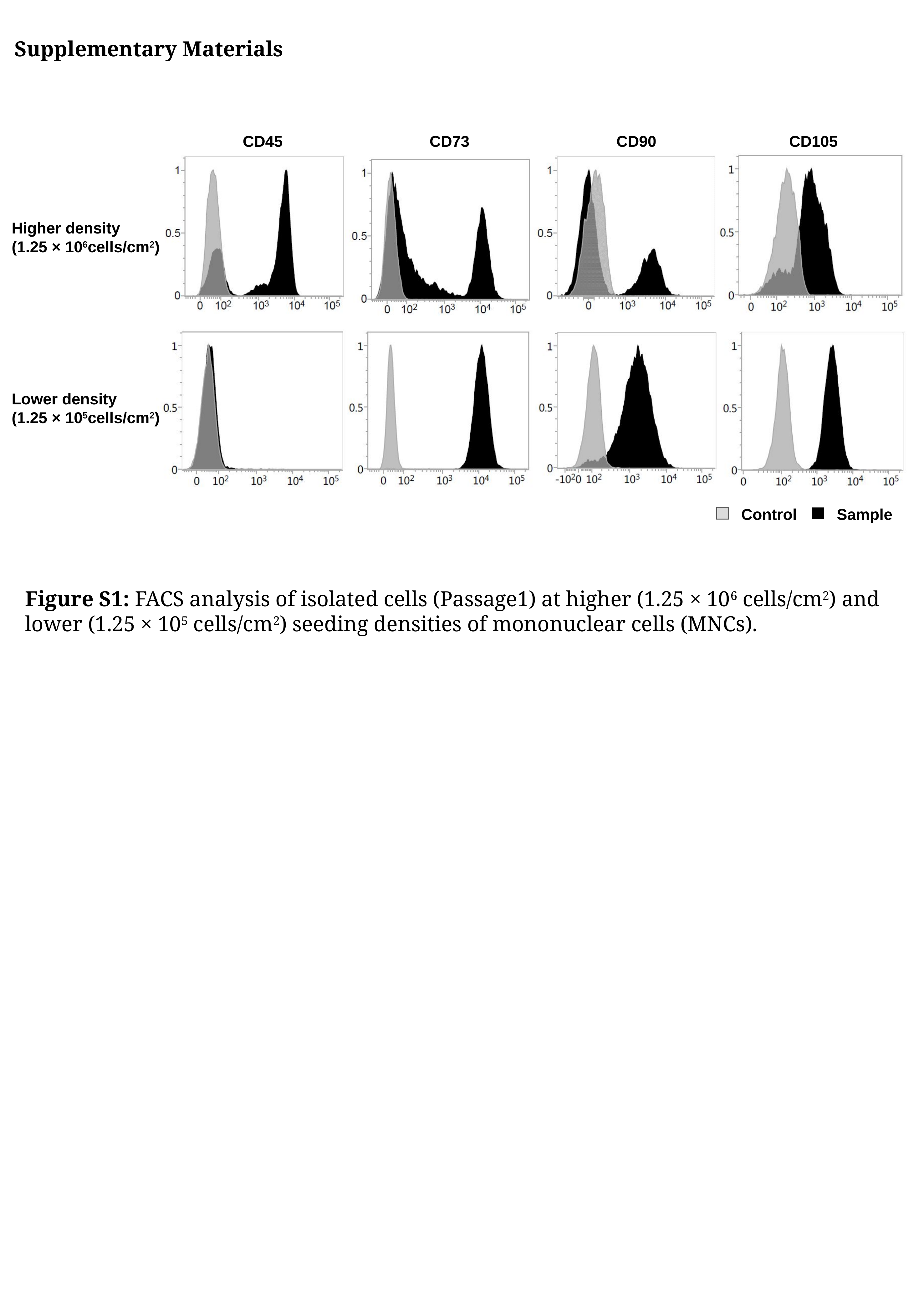

Supplementary Materials
CD45
CD73
CD90
CD105
Higher density
(1.25 × 10^6^cells/cm2)
Lower density
(1.25 × 10^5^cells/cm2)
Control
Sample
Figure S1: FACS analysis of isolated cells (Passage1) at higher (1.25 × 10^6^ cells/cm2) and lower (1.25 × 10^5^ cells/cm2) seeding densities of mononuclear cells (MNCs).

## Slide 2
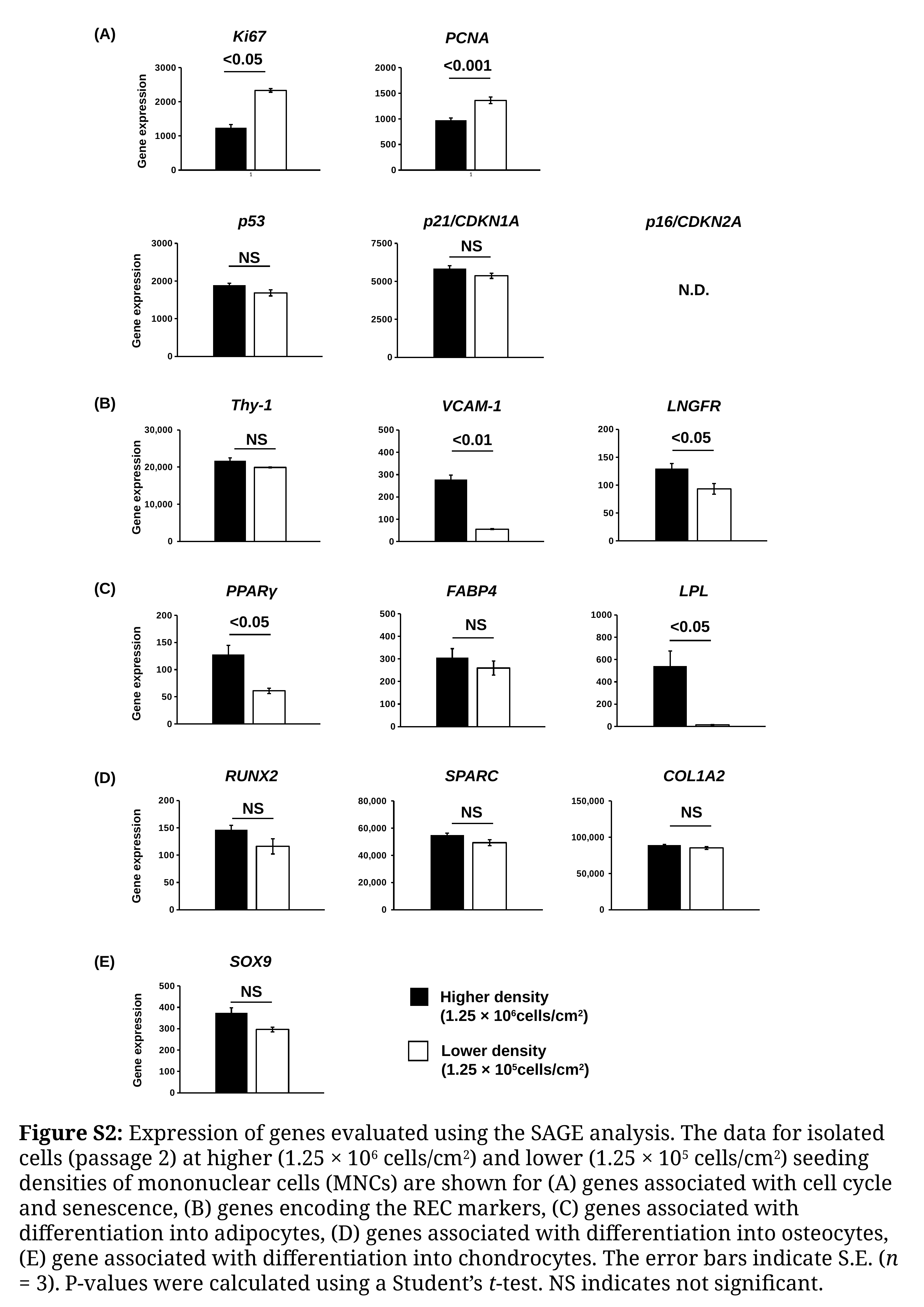

(A)
Ki67
PCNA
<0.05
<0.001
### Chart
| Category | High density | Low density |
|---|---|---|
### Chart
| Category | High density | Low density |
|---|---|---|Gene expression
p53
p21/CDKN1A
p16/CDKN2A
NS
### Chart
| Category | High density | Low density |
|---|---|---|
### Chart
| Category | High density | Low density |
|---|---|---|NS
N.D.
Gene expression
(B)
Thy-1
VCAM-1
LNGFR
### Chart
| Category | High density | Low density |
|---|---|---|
### Chart
| Category | High density | Low density |
|---|---|---|
### Chart
| Category | High density | Low density |
|---|---|---|<0.05
NS
<0.01
Gene expression
(C)
PPARγ
FABP4
LPL
### Chart
| Category | High density | Low density |
|---|---|---|
### Chart
| Category | High density | Low density |
|---|---|---|
### Chart
| Category | High density | Low density |
|---|---|---|<0.05
NS
<0.05
Gene expression
RUNX2
SPARC
COL1A2
(D)
### Chart
| Category | High density | Low density |
|---|---|---|
### Chart
| Category | High density | Low density |
|---|---|---|
### Chart
| Category | High density | Low density |
|---|---|---|NS
NS
NS
Gene expression
(E)
SOX9
### Chart
| Category | High density | Low density |
|---|---|---|NS
Higher density
(1.25 × 10^6^cells/cm2)
Gene expression
Lower density
(1.25 × 10^5^cells/cm2)
Figure S2: Expression of genes evaluated using the SAGE analysis. The data for isolated cells (passage 2) at higher (1.25 × 10^6^ cells/cm2) and lower (1.25 × 10^5^ cells/cm2) seeding densities of mononuclear cells (MNCs) are shown for (A) genes associated with cell cycle and senescence, (B) genes encoding the REC markers, (C) genes associated with differentiation into adipocytes, (D) genes associated with differentiation into osteocytes, (E) gene associated with differentiation into chondrocytes. The error bars indicate S.E. (n = 3). P-values were calculated using a Student’s t-test. NS indicates not significant.

## Slide 3
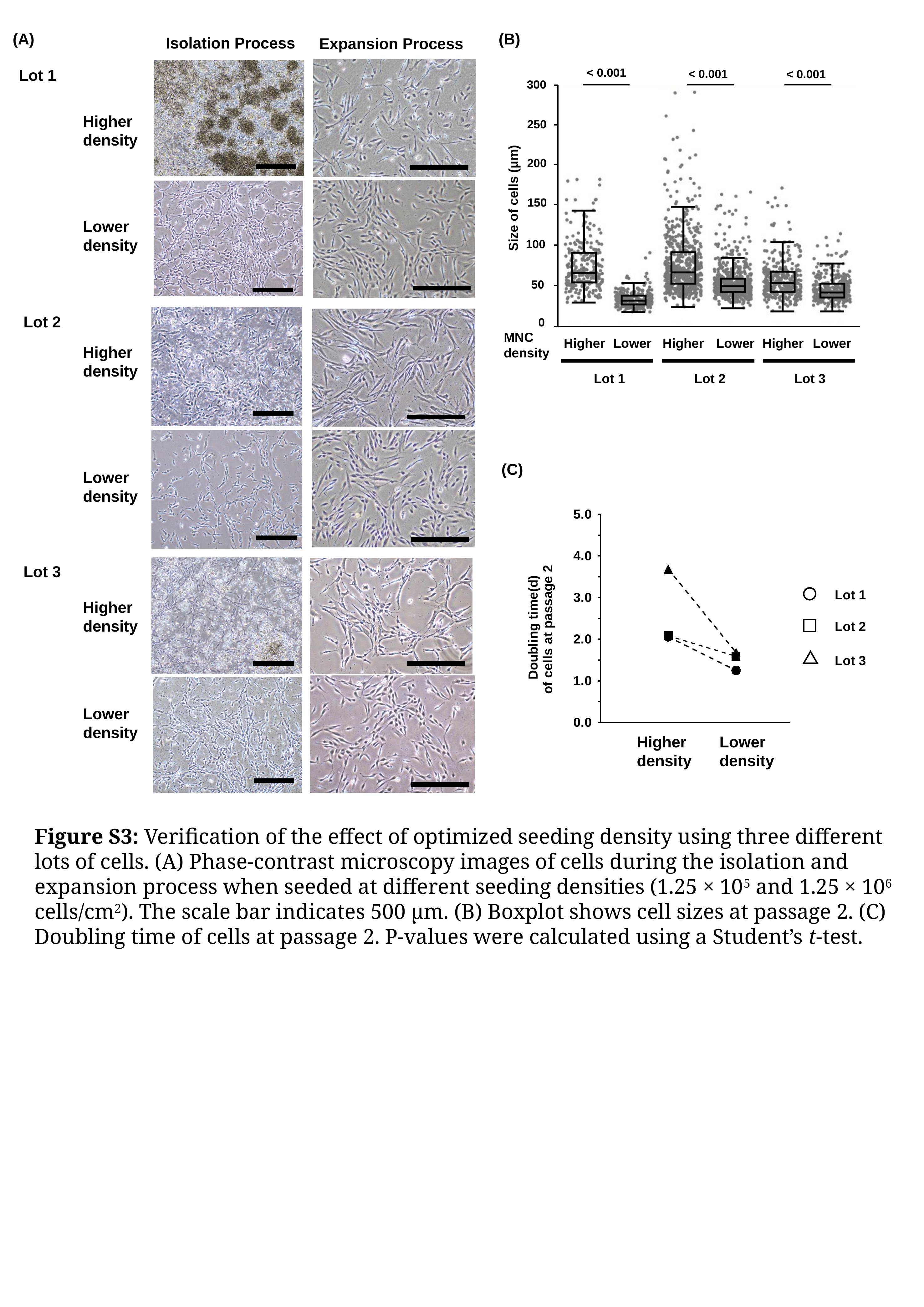

(A)
(B)
Isolation Process
Expansion Process
< 0.001
Lot 1
< 0.001
< 0.001
300
250
200
Size of cells (µm)
150
100
50
0
Higher
density
Lower
density
Lot 2
MNC
density
Higher
Lower
Higher
Lower
Higher
Lower
Higher
density
Lot 1
Lot 2
Lot 3
(C)
Lower
density
### Chart
| Category | | | |
|---|---|---|---|
Lot 3
Lot 1
Lot 2
Lot 3
Higher
density
Lower
density
Higher
density
Lower
density
Figure S3: Verification of the effect of optimized seeding density using three different lots of cells. (A) Phase-contrast microscopy images of cells during the isolation and expansion process when seeded at different seeding densities (1.25 × 10^5^ and 1.25 × 10^6^ cells/cm2). The scale bar indicates 500 µm. (B) Boxplot shows cell sizes at passage 2. (C) Doubling time of cells at passage 2. P-values were calculated using a Student’s t-test.
